# Supplementary material for: Mothers’ sleep deficits and cognitive performance: Moderation by stress and age
Source: PLoS One. 2021 Jan 7;16(1):e0241188. doi: 10.1371/journal.pone.0241188 (PMC7790244; doi:10.1371/journal.pone.0241188)
Supplement: Attachment — Submitted filename: Response to Reviewers_PlosOne_R1.docx [file pone.0241188.s002.docx]

**Comments from Editor:**

1.Please ensure that your manuscript meets PLOS ONE's style requirements, including those for file naming. The PLOS ONE style templates can be found at
<http://www.journals.plos.org/plosone/s/file?id=wjVg/PLOSOne_formatting_sample_main_body.pdf> and <http://www.journals.plos.org/plosone/s/file?id=ba62/PLOSOne_formatting_sample_title_authors_affiliations.pdf>

**We have made the necessary changes to section headings, tables, figures, and references.**

2. Please change "Caucasian” to “White” or “of [Western] European descent” (as appropriate).

**We have made this change. The text now reads “Ninety percent of mothers were White, 4% were Hispanic, 3% were African American, 1% were Asian American, and 2% identified as mixed race, American Indian, or other.” See page 9, line 178.

“Also, 90% of the mothers were White, rendering our results less generalizable to non-White samples” See page 24, lines 521-522.**

3. Thank you for stating the following in the Acknowledgments Section of your manuscript:
"The study has been funded by Grants MH099437 from the National Institute of Mental Health and HD073202 from the Eunice Kennedy Shriver National Institute of Child Health and Human Development."

We note that you have provided funding information that is not currently declared in your Funding Statement. However, funding information should not appear in the Acknowledgments section or other areas of your manuscript. We will only publish funding information present in the Funding Statement section of the online submission form.

Please remove any funding-related text from the manuscript and let us know how you would like to update your Funding Statement. Currently, your Funding Statement reads as follows:
"NO The funders had no role in study design, data collection and analysis, decision to publish, or preparation of the manuscript."

**We have removed the funding-related text from the Acknowledgements section of the manuscript. Instead, we have added this information to the Funding Statement section of the online submission form.**

4. Please include your tables as part of your main manuscript and remove the individual files. Please note that supplementary tables (should remain/ be uploaded) as separate "supporting information" files.

**We have included tables as part of the main manuscript. See pages 17-20.**

**Comments from Reviewer:**

1. The hypotheses are centered on the possibility that sleep and stress may additively supplement or multiplicatively moderate one another. However, it is not clear how the authors tested their first hypothesis of the “additive effects” of stress and sleep. Are the authors referring to the main effects included in Step 1? This approach would test the effects of each predictor (when covarying for the other predictor), whereas adding standardized values of the predictors may better reflect addictive effects if this is the primary hypotheses.

**We were referring to the main effects in Step 1. To clarify, we have removed the word “additive” from the manuscript (in multiple locations), including the hypothesis 1 wording. By “additive” we meant whether sleep and stress provided “independent” prediction of multiple predictors; therefore we have replaced “additive” with “independent” throughout the manuscript (to distinguish it from a non-independent “interactive” or “moderation” effect). We also clarified (p. 18, lines 391-394) that we included the products of standardized predictors, to test interaction effects.

2. With the cross-sectional study design, it is impossible to know the directionality of the proposed associations and whether cognitive functioning is a predictor of poor sleep, particularly among mothers with more stress. For instance, mothers with poorer cognitive functioning may have more difficulty regulating stress in their lives (particularly when they experience heightened levels), which may contribute to poor sleep. This possibility warrants further discussion.

**We agree that the cross-sectional design does not allow us to test this hypothesis. We have added the limited scope of analyses with cross-sectional data as a limitation in the discussion (p. 23, lines 511-514), and point out that a different temporal pattern like that suggested by the reviewer, could be operating.**

3. The rationale for age as the moderator of interest related to stress and sleep in predicting cognitive functioning is unclear. As the authors discuss, the ages of mothers with similarly aged toddlers are both (theoretically) linked to cognitive performance and several of the stress indicators (e.g., education, SES). Particularly since the authors do not have clear hypotheses for age, what is the practical or theoretical significance for examining age in these associations? It would be helpful to provide more information highlighting the importance of examining age as a moderator.

**Adding age as a moderator allowed us to examine whether the link between sleep, stress and cognitive performance would operate differently for older vs. younger mothers. Since there is literature showing that age is associated with sleep differences (e.g., declines in slow wave sleep and total duration of sleep; Hall et al., 2015), and because age is associated with differences in parental stress (younger mothers often have less education and financial security; older mothers report less conflict with children and report feeling more competent when dealing with childrearing stress; Barnes 2006; Barnes, Gardiner, Sutcliffe & Melhuis, 2013), adding age as a moderator would allow us to see if the relationship between sleep and stress on cognitive performance was dependent on mother age. We have added this information in the introduction section. See pages 6-7, lines 130-134.**

4. Similarly, it is surprising that certain factors were not considered or covaried in the present study, such as number of other children in the household and occupation status. This seems particularly likely to contribute to additional parental stressors, difficulty managing role overload, SES, and/or poorer sleep.

**In the McQuillan et al 2019 paper we cite, the number of children as well as number of hours worked outside the home were included as covariates in the relationship between stress and parenting. It was found that mothers’ sleep was associated with stress, even with these covariates accounted for, so we did not include it in the stress composite in the current paper. As for occupation status, occupational prestige is included in the Hollingshead SES score that we used for the study. **

5. There is little explanation for why the specific sleep domains of interest were selected and how they may be similarly/differently associated with cognitive performance (particularly in interaction with stress/age). Some of this is discussed later in the manuscript, and would be more helpful in the introduction.

**Most research on sleep effects on cognitive performance have looked at sleep deprivation, i.e., short durations of sleep. There is research showing that aging is related to decreases in total duration of sleep and more nighttime activity during sleep. There is also work showing that daily stress is related to more sleep variability and shorter durations of sleep. We have added these studies to the introduction to rationalize the use of our sleep components. See pages 4-5, lines 84-98).

6. Although the cumulative risk index was used in the authors’ prior study, there are several indicators that are more strongly related (e.g., SES, household status, role overload, etc) and on varying time scales (e.g., daily hassles and SES). How is the CRI calculated and has it been validated as a measure of chronic stress with these indices? For instance, it is also surprising that the CBCL externalizing symptoms scale is included.

**We chose the stressors we used for the CRI because they were comparable to the three domains of the widely-used Parenting Stress Index (Abidin, 1990): feelings of distress, difficult child behavior, and dysfunctional parent-child interactions. Also though there may not be consensus about which stress variables should be included for a cumulative measure of stress, previous research has shown that the precise combination of stressors is not as important compared to the choice to consider risk in aggregate (Sameroff et al., 1993; Evans, Whipple & Li, 2013).

The CBCL was included since there is research showing that mother perceptions of high levels of child aggression can be a significant stressor (Miragoli et al., 2018, *Child Abuse & Neglect*).**

7. The use of PCA for actigraphic sleep is an interesting approach, and utilizes more of the actigraphic data. However, it is unclear how these latent domains are similar to the widely-used and validated sleep characteristics typically derived from actigraphy?

** Sleep efficiency is one commonly used actigraphy variable used in sleep research. However, we chose not to use it for several reasons. We argue that our composite variables expand on the sleep efficiency variable because they incorporate more information. For example, our sleep activity composite includes the average time awake after sleep onset, the average minute-by-minute activity level, the average number of night awakenings lasting at least five minutes, the average duration of the longest wake episode after sleep onset, and the average percent of active epochs after sleep onset. Notably, in our data, our sleep activity composite is strongly correlated with the actigraphic sleep efficiency variable, *r*= - 0.94, such that more activity in the night reflects less efficient sleep, as expected.

The sleep timing composite similarly includes more information as it is comprised of average time of midsleep, average time of sleep onset, and average bedtime.

Given the high correlation between the actigraphic indexes, our findings with the sleep composites can be interpreted as findings pertaining to sleep efficiency, but our usage of the composite variable also allows our findings to be interpreted relative to other variables that are commonly used in the sleep literature. Additionally, one issue with using the sleep efficiency variable alone (as pointed out by Meltzer, Montgomery-Downs, Insana, and Walsh, 2012), is that there is little to no consensus in the field about precisely how this variable should be computed. In fact, only about 65% of the studies reviewed by Meltzer and colleagues that used “sleep efficiency” reported how this variable was defined or computed. For sleep efficiency, one could use the ratio of time asleep over the sleep period from sleep onset to offset, *or* one could use the ratio of time asleep over the down period/period of time in bed from diary reported downtime and uptime. Without consensus in the field, we opted to analyze our data with the more generalizable, but comparable, composite indices. Our four sleep composites—sleep duration, sleep timing, sleep variability, and sleep activity—represent broad dimensions of actigraphy that are often examined in the sleep literature (Meltzer et al., 2012).

Finally, the concern we would have with using only one or two indicators for each of the major sleep dimensions is that they are not as reliable as they could be; making full use of all available data from actigraphy and diaries, addresses this measurement concern.

Published papers that have used our sleep composites include:

1. Staples, A. D., Bates, J. E., Petersen, I. T., McQuillan, M. E., & Hoyniak, C. (2019). Measuring sleep in young children and their mothers: Identifying actigraphic sleep composites. *International Journal of Behavioral Development*, *43*(3), 278-285.
2. Hoyniak, C. P., Bates, J. E., Staples, A. D., Rudasill, K. M., Molfese, D. L., & Molfese, V. J. (2019). Child sleep and socioeconomic context in the development of cognitive abilities in early childhood. *Child Development*, *90*(5), 1718-1737.
3. McQuillan, M. E., Bates, J. E., Staples, A. D., & Deater-Deckard, K. (2019). Maternal stress, sleep, and parenting. *Journal of Family Psychology, 33*(3), 349-359.
4. Chary, M., McQuillan, M. E., Bates, J. E., & Deater-Deckard, K. (2020). Maternal Executive Function and Sleep Interact in the Prediction of Negative Parenting. *Behavioral Sleep Medicine*, *18*(2), 203-216.
5. Hoyniak, C.P., Bates, J.E., McQuillan, M.E., Staples, A.D., Petersen, I. T., Rudasill, K.M., & Molfese, V.J. (2020). Sleep across early childhood: Implications for internalizing and externalizing problems, socioemotional skills, and cognitive and academic abilities in preschool. *Journal of Child Psychology and Psychiatry.* doi: 10.1111/jcpp.13225
6. Hoyniak, C.P., Bates, J. E., McQuillan, M.E., Albert, L. E., Staples, A.D., Molfese, V.J., Rudasill, K.M., & Deater-Deckard, K. (under review). The family context of toddler sleep: Routines, sleep environment, and emotional security induction in the hour before bedtime.
7. McQuillan, M. E., Bates, J. E., Staples, A. D., Hoyniak, C. P., Rudasill, K.M., & Molfese, V. J. (in preparation). Sustained attention across toddlerhood: The roles of language and sleep.
8. Hoyniak, C.P., McQuillan, M.E., Bates, J. E., Staples, A. D., Schwichtenberg, A.J., & Honaker, S.M. (in preparation). Objective and subjective pre-sleep arousal and sleep in early childhood.**
8. What is the rationale for dichotomizing continuous variables? For instance, age is dichotomized into two groups of under/over 32, which artificially creates two groups. Further, sleep variables are dichotomized into subgroups with low and high sleep activity/timing rather than applying a median split as used for other variables.

**Median splits were used for all the variables including sleep to facilitate analysis using ANOVAs, which are simply a special case of the general linear model. We chose to use ANOVAs in order to more simply represent the more complex, higher-order interaction effects.**

9. What was the process for scoring actigraphy? For instance, how did these scoring metrics compare to those reported by Patel and colleagues (2015) to enhance reproducibility of actigraphy scoring?

**Whenever possible, mothers’ sleep diaries were used to inform and enhance data obtained from actigraphs, such as end of rest period. All mother actigraph data were scored with the adult-validated Cole-Kripke algorithm ([Cole, Kripke, Gruen, Mullaney, & Gillin, 1992](https://www.ncbi.nlm.nih.gov/pmc/articles/PMC6822101/#R8)). Periods when the actigraph was not worn or when sleep occurred while moving, such as in a car, were excluded from sleep scoring so that sleep minutes would not be overestimated or underestimated.

Sleep was scored during the period between the diary reported bedtime (or nap time) and the first epoch for which the actigraphic activity count reached 50 and remained above that threshold until the next sleep interval. Minutes asleep while in bed were based on the bedtime reported in the diary and actigraphic measure determined end of sleep. Variables concerning activity and awakenings after sleep onset were based on motion recordings by the actigraph using the zero-crossing mode and a moderate sensitivity threshold, which is the commonly used method in sleep research (Meltzer, Montgomery-Downs, Insana, & Walsh, 2012). A night waking was scored when the activity count was above threshold (50 crossings) for at least five minutes. The actigraph variables considered in this study were consistent with those variables used in prior research with adults ([Berger et al., 2008](https://www.ncbi.nlm.nih.gov/pmc/articles/PMC6822101/#R6); [Meltzer et al., 2012a](https://www.ncbi.nlm.nih.gov/pmc/articles/PMC6822101/#R21)).**

10. For the daily hassles measure, how was this score computed? Was the stressor weighted by intensity or were these summed for a total of intensity and frequency?

**The Parenting Daily Hassles Questionnaire is comprised of 20 items that yield two subscales: challenging child behavior (7 items) and parenting tasks (8 items). In this paper, we summed the mothers’ ratings of frequency of the eight parenting tasks (1 = never to 4 = constantly; range : 0- 32*)* as well as her ratings’ of the intensity of the hassle (1 = no hassle to 5 = big hassle; range: 0 – 40) for those eight tasks. Both ratings were summed together for the final score.

We have added this information to the manuscript (p. 11, lines 230-233).**

11. How do the norms of cognitive performance tests in the current study compare to those in the general population? Were the cognitive tasks normed for age and gender?

**For the Shipley test, raw scores were used because mother age was used as a predictor in the equation. Using the age-normed scores would remove the variance due to age in the scores, and also render interpretation of any estimated interaction with age uninterpretable. However, in our sample, for the age-normed scores, *M* = 105.47, *SD* = 9.48. This is very close to the population average of 100, *SD* = 15 (Mason, C. F., & Ganzler, H. (1964). Adult norms for the Shipley Institute of Living Scale and Hooper Visual Organization Test based on age and education. *Journal of Gerontology*, *19*(4), 419-424.)

Also, the mother EF tasks do not have published age norms (although we now include, in the Method section, a note that the distributions on the EF tasks were similar to those of other studies of adult women in community samples, see page 15, lines 322-324). Therefore, we used non-normed scores for both cognitive tasks.**

12. How did mothers who were included in the current study compare to those eliminated with missing data? It seems that there are 314 in the prior published study with same sample.

**The reason the McQuillan 2019 paper has a larger sample is because they used data from a pilot study they conducted separately in Bloomington, IN (this study was done to demonstrate feasibility before the multi-site study began). In that study, mothers did not complete all of the questionnaires used in this study nor did they complete the executive function tasks, so we did not include the pilot study mothers in our analysis.**
