# Supplementary material for: Mothers’ sleep deficits and cognitive performance: Moderation by stress and age
Source: PLoS One. 2021 Jan 7;16(1):e0241188. doi: 10.1371/journal.pone.0241188 (PMC7790244; doi:10.1371/journal.pone.0241188)
Supplement: Attachment — Submitted filename: Response to Reviewers_PLosOne_R3.docx [file pone.0241188.s003.docx]

**Editor Comments:**

1) I appreciate the challenges of interpreting three-way interactions when those interactions are based on continuous variables. However, methods are present to visualize and interpret the data without truncating much information. For example, <http://www.jeremydawson.co.uk/slopes.htm>, <https://tomhouslay.com/2014/03/21/understanding-3-way-interactions-between-continuous-variables/>, or (perhaps most useful) <https://cran.r-project.org/web/packages/interactions/vignettes/interactions.html>. Frankly, I do not think that the implementation matters; however, to strengthen the conclusions that you are presenting, relying on the data, rather than dichotomizing the continuous predictors.

At a minimum, providing support for the three-way interaction using the continuous variables is necessary. That could provide enough support for the approach taken to visualize the results.

**We appreciate the editor’s emphasis on using continuous variables wherever possible. However, there are a few reasons why we were unable to use this method for our analyses:

a) While there are certainly templates online (such as the links provided by the editor) to visualize 3-way interactions, all of the formats dichotomize at least one of the moderators into two groups and graph out the interaction effect between the continuous independent variable and the continuous dependent variable as a function of the continuous proximal moderator. There is no way that we know of that allows us to present a graph with all three of the variables remaining continuous. As quoted on the [DataScience website](https://datascienceplus.com/interpreting-three-way-interactions-in-r/) regarding graphing interaction effects, “Three-way interactions between continuous variables create a 4D surface between all continuous variables and the response variable. *[sic]* Human mind cannot grasp 4D surface so we have to rely on simplifications (similar in ways to what can be done for two-way interactions) to explore three-way interactions."
b) The other reason why we chose to not use the templates provided in this review is because in a previous review letter, the editor had suggested using the Johnson-Neyman technique to identify regions of significance in interaction effects. This technique is the most robust method to leverage the value of continuous variables. In the graphs (figures 1a, 1b, 2a, and 2b) the lines represent the effect size of the relationship between the independent and dependent variable at various levels of the moderator. This way, instead of examining just the means as one would with a categorical variable, we are taking into account how the association between the two continuous variables changes along values of the continuous moderator.

In sum, we agree with the editor that we need to leverage the value of continuous variables. Upon further reflection, we agree that the Johnson-Neyman technique is thus far, the most accurate and rigorous way to probe the post-hoc analyses of a three-way interaction. Thus, we have chosen to re-analyze all of the interaction effects using this method as opposed to the ANOVA we had previously used. We found that the pattern of results remained the same when using the Johnson-Neyman plots for sleep activity. For sleep timing however, there was a change in which regions were significant. We find now that sleep timing interacts with stress and age on cognition only among younger women. The results are explained in detail in our results section (see page 19) and our discussion (see pages 21-22).**

2) The supplementary file with the full dataset is very helpful. However, some identification of the continuous and dichotomous variables used in the main analyses would be critical for the spirit of data sharing and transparency.

**We have attached a dataset that only includes the variables used in this paper. The column “Label” explains what each variable is.**
